# A ketamine package for use in emergency cesarean delivery when no anesthetist is available: An analysis of 401 consecutive operations

**DOI:** 10.1002/ijgo.13965

**Published:** 2021-10-28

**Authors:** Thomas F. Burke, Sreekar Mantena, Kennedy Opondo, Solomon Orero, Khama Rogo

**Affiliations:** ^1^ Global Health Innovation Laboratory Department of Emergency Medicine Massachusetts General Hospital Boston Massachusetts USA; ^2^ Harvard Medical School Boston Massachusetts USA; ^3^ Harvard T.H. Chan School of Public Health Boston Massachusetts USA; ^4^ Departments of Statistics and Molecular & Cellular Biology Harvard University Cambridge Massachusetts USA; ^5^ African Institute for Health Transformation Sagam Community Hospital Luanda Kenya; ^6^ Kenya Obstetrics and Gynecological Society Nairobi Kenya

**Keywords:** anesthesia, cesarean delivery, maternal health, resource‐limited setting

## Abstract

**Objective:**

To evaluate the safety and effectiveness of a ketamine‐based anesthesia package to support emergency cesarean section when no anesthetist is available.

**Methods:**

A prospective case‐series was conducted between December 11, 2013 and September 30, 2021 across nine sub‐county hospitals in Kenya. Non‐anesthetist healthcare providers undertook an evidence‐based five‐day training course. A structured instrument was used to collect preoperative, intraoperative, and postoperative data, and patients were contacted 6 months following the surgery to collect outcomes. The primary outcome measures were maternal and newborn survival and the ability of the ketamine package (ESM‐Ketamine) to safely support cesarean deliveries.

**Results:**

A total of 401 emergency cesarean sections were performed using ketamine, administered by 54 non‐anesthetist providers. All mothers survived to discharge. Brief oxygen desaturations were recorded among 33 (8.2%) mothers, and agitation and hallucinations occurred among 13 (3.2%). There were no maternal serious adverse events. At 6‐month follow‐up, 94.2% of mothers who could be reached reported no complaints. Additionally, 402 (92.4%) of the 435 operative births survived to discharge.

**Conclusion:**

The ESM‐Ketamine package can be used by trained non‐anesthetist providers to support emergency cesarean sections when no anesthetist is available. Ketamine has significant potential to increase access to emergency cesarean deliveries in resource‐limited settings.

## Abstract 2

A ketamine‐based anesthesia package was found to be safe and effective in support of 401 emergency cesarean sections when used by trained non‐anesthetist healthcare providers.

## 1 INTRODUCTION

Approximately 300 000 mothers die and many more are seriously injured from pregnancy‐related causes each year worldwide.^1^ While maternal mortality has significantly decreased over the past two decades, it is still unacceptably high in resource‐poor settings. In 2017, high‐income countries had an average maternal mortality ratio of 11 deaths per 100 000 live births, while the average for low‐income countries was over 40 times greater, at 462 per 100 000 births.^1^ Emergency and essential surgical care, such as cesarean delivery, is vital to reducing the burden of maternal mortality, but often remains inaccessible in resource‐poor settings.^2^ Although the World Health Organization (WHO) considers the ideal rate for cesarean sections to be between 10%–15%, a recent study found that the poorest quintile of countries have a mean rate of only 3.7%.^3^, ^4^ The severe shortage of anesthetists across resource limited settings has been identified as a major barrier to providing these emergency operative procedures.^5^

The anesthesia workforce density is 100 times greater in high‐income countries than in low‐income countries, and recent surveys of referral hospitals in Africa found that only 7% have adequate anesthesia staff.^6^, ^7^ Many low‐resource countries are implementing anesthetist training programs, but a 2014 systematic review found that even the most ambitious expansions would fall significantly short of meeting the global anesthesia gap in the coming decades.^5^ Novel solutions are urgently needed to combat the anesthesia crisis.

Ketamine is a WHO essential medicine and has been used for over five decades to provide safe anesthesia and analgesia. It has a wide safety profile, does not depress breathing or lower blood pressure, and does not require extensive patient‐monitoring equipment.^8^ It is widely used to provide surgical anesthesia in conflict zones and disaster situations when no anesthetists are available, but its use in maternal care remains limited.^9^ We designed, tested, and implemented a novel ketamine‐based anesthesia program to address the anesthesia gap in Kenya. The ‘Every Second Matters for Emergency and Essential Surgery‐Ketamine’ (ESM‐Ketamine) package was originally designed to support emergency cesarean sections and other reproductive health operations when no anesthetist is available. Out of identified need, the ESM‐Ketamine providers expanded the use cases to general surgery, orthopedic surgery, and head/neck procedures among other indications, which have been described in prior work.^10^, ^11^ In this study, we specifically examined the use of ESM‐Ketamine to support emergency cesarean section when no anesthetist was available and evaluated its effectiveness and safety by analyzing maternal and newborn outcomes.

## 2 MATERIALS AND METHODS

**FIGURE 1:**
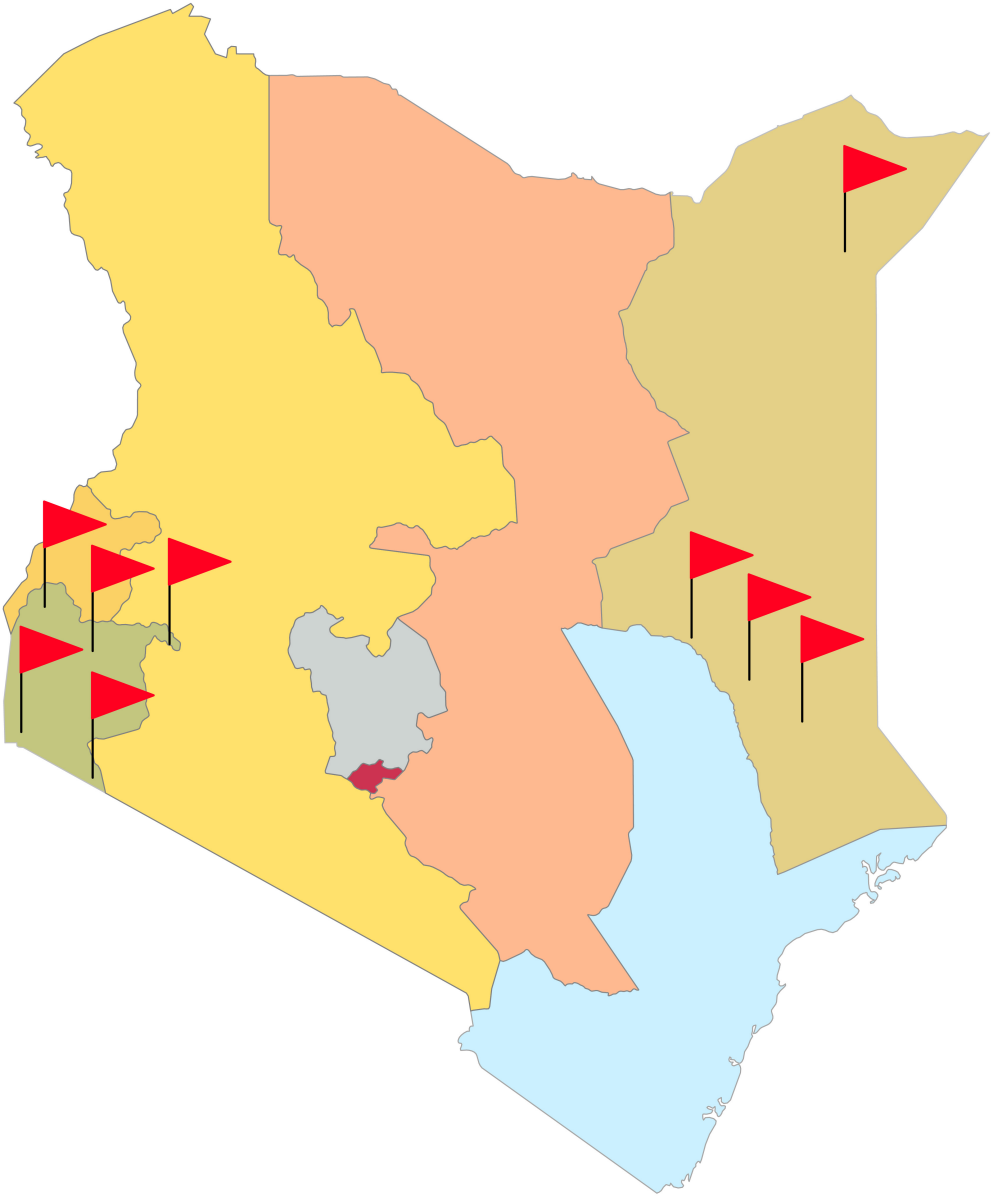
Location of facilities using ESM‐Ketamine package of emergency cesarean delivery

Data was collected prospectively from seven sub‐county and two private hospitals in Kenya. The locations of these facilities are depicted in Figure 1, and the characteristics of these facilities are presented in Table S1. The rationale for selecting these nine study sites has been previously described.^10^ The Sagam Community Hospital implemented the ESM‐Ketamine program first, doing so on December 11, 2013, and the Kendu Sub‐County hospital was the last to implement the program, doing so on September 15, 2019 (Table 1). All data until September 30, 2021 were considered in this study.

**TABLE 1:** Location of included emergency operative procedures

Hospital name	Hospital county	ESM‐Ketamine‐assisted cesarean deliveries (*n*, %)	Date of first ESM‐Ketamine case at hospital	Number of on‐line months of ESM‐Ketamine program
Sagam Community Hospital	Siaya County	204 (50.9%)	12/11/2013	112
Maseno Mission Hospital	Kisumu County	40 (10.0%)	7/21/2017	29
Muhoroni Sub‐County hospital	Kisumu County	19 (4.7%)	5/10/2019	17
Kendu Sub‐County Hospital	Homa Bay County	51 (12.7%)	9/15/2019	24
Mbita Sub‐County Hospital	Homa Bay County	21 (5.2%)	5/8/2015	20
Dadaab Sub‐County Hospital	Garissa County	3 (0.7%)	11/29/2016	10
Modogashe Sub‐County Hospital	Garissa County	2 (0.5%)	12/1/2016	9
Ijara Sub‐County Hospital	Garissa County	55 (13.7%)	3/29/2017	33
Takaba Sub‐County Referral Hospital	Mandera County	6 (1.5%)	5/7/2016	13

When on‐line with ESM‐Ketamine, study hospitals had a functional operating theater, available perioperative services, a staff clinician able to perform emergency cesarean delivery and other emergency procedures, but limited access to anesthesia services. The study protocol was approved by the Kenyan Ministry of Health and the institutional review boards of Partners Healthcare and the Maseno University School of Medicine. These two institutional review boards, the Division of Reproductive Health of the Kenya Ministry of Health, the leaders of the participating hospitals, and the County Health Directors provided approval for the nurse‐midwives, clinical officers (mid‐level non‐physician providers), medical officers (generalist medical doctors who completed medical school and one year of an internship), and non‐anesthetist physicians to become ESM‐Ketamine providers.

A detailed description of the development and implementation of the ESM‐Ketamine program has been reported elsewhere.^11^ The package includes an ESM‐Ketamine kit, an intensive 5‐day training program for trainees, checklists, and wall charts. All trainees for the ESM‐Ketamine program were non‐anesthetist healthcare providers such as nurse‐midwives, clinical officers, and medical officers. The 5‐day training course educated providers on the basic pharmacology of ketamine, contraindications, dosing schedule, patient monitoring, and essential ventilation support for both adults and newborns. Additional training on the use and dosing schedule of supplemental medications to control adverse effects included diazepam for agitation or hallucinations, hydralazine for severely elevated blood pressure, atropine for hypersalivation, and promethazine and prochlorperazine for nausea and vomiting. Each ESM‐Ketamine provider was also fully trained in Helping Babies Breathe. The skills of providers were reinforced through hands‐on case‐based simulation followed by mentored procedures in the operating theater.

According to the protocol, when the need for an emergency cesarean section was identified at a participating site, attempts were first made to contact a trained anesthetist. If no anesthesia providers were available after multiple attempts and safe transfer to another facility was not possible, the ESM‐Ketamine protocol was activated. During each of the ESM‐Ketamine‐supported cesarean sections, the overall responsibility for the patient was held by the surgical provider who performed the operation. The ESM‐Ketamine provider administered the anesthetic and monitored the patient intraoperatively and during recovery. Preoperative, intraoperative, and postoperative data as well as discharge conditions were collected from all patients using a structured instrument. To record maternal and newborn outcomes at 6 months, attempts were made to contact all enrolled mothers. If the cesarean section occurred during the last 6 months of the study period, the final follow‐up was in less than 6 months. A standardized script was used to verify the mothers’ identity and date of the cesarean procedure, and mothers were surveyed about their own health and that of the newborn. To ensure all maternal and newborn deaths and their causes were identified, hospital records and county maternal, newborn, and stillborn death audits were reviewed independently of the ESM‐Ketamine providers and implementing teams.

All ESM‐Ketamine‐supported emergency cesarean deliveries conducted within the participating facilities were extracted from the database for analysis. The primary outcome measures were maternal and newborn survival and the ability of ESM‐Ketamine to safely support cesarean deliveries. Adverse events were defined as hallucinations or agitation treated with diazepam, hypersalivation treated with atropine, peripartum hypertension treated with hydralazine, and brief periods of oxygen desaturations (SpO_2_ < 92% for <30 s). Serious adverse events were defined as maternal death, prolonged periods of oxygen desaturations (SpO_2_ < 92% for >30 s), and any bag‐valve mask ventilation support. Other variables collected included facility demographics, maternal age and weight, total ketamine dose administered, length of procedure, and notes from 6‐month follow‐up calls.

The database was constructed using Excel 2015 (Microsoft, Redmond, WA, USA). Data were analyzed using Python 3.7. Standard descriptive and frequency analyses were performed. A chi‐squared test of independence was used to compare newborn mortality rates between ESM‐Ketamine provider types (nurse‐midwives, medical officers, and clinical officers). A *P*‐value of <0.05 was considered significant.

## 3 RESULTS

Over a 7‐year period, 54 ESM‐Ketamine trained providers from nine healthcare facilities in Kenya used the ESM‐Ketamine package to support 401 emergency cesarean deliveries when no anesthetist was available. All cesarean sections performed at the nine facilities during the study months were included. No cases supported by the ESM‐Ketamine package were excluded. The nine facilities were on‐line with the ESM‐Ketamine program for a combined total of 204 facility‐months.

The majority of the emergency cesarean sections (50.9%) were performed at Sagam Community Hospital, and six facilities performed more than 10 operative procedures each (Table 1). The average ketamine dose administered per cesarean section was 371 ± 201 mg, and the mean duration of the operative procedures was 55 ± 26 min.

The mean age and weight of mothers whose cesarean sections were supported by the ESM‐Ketamine package were 26.2 ± 7.5 years and 68.6 ± 10.9 kilograms, respectively (Table 2). Among the 401 mothers, 34 (8.5%) had twin pregnancies, while the rest had singleton pregnancies.

**TABLE 2:** Demographic characteristics of mothers who received the ESM‐Ketamine package for emergency cesarean delivery

Characteristic	Mean ± SD or *n*, %
Age, mean (years)	26.2 ± 7.5
10–19	66 (16.5%)
20–29	224 (55.9%)
30–39	97 (24.2%)
≥40	14 (3.5%)
Weight, mean (kg)	68.6 ± 10.9
Singleton pregnancies	318 (91.5%)
Twin pregnancies	34 (8.5%)

All 401 mothers who received ESM‐Ketamine survived their cesarean section and were discharged with no complaints (Table 3). There were no reported maternal serious adverse events, and none of the procedures required resuscitation or bag‐valve mask ventilation of the mother. Brief oxygen desaturations (<92% for <30 s) were observed among 34 (8.5%) mothers (Table 4). Hallucinations and agitation, treated with diazepam, were observed in 13 (3.2%) mothers. Additionally, seven (1.7%) mothers were treated with hydralazine for hypertension of pregnancy and 63 (15.7%) mothers were treated with atropine for hypersalivation. Of the 401 mothers, 294 (73.3%) could be reached for follow‐up. For 46 of these 294 mothers (15.6%), their operations fell within the final 6 months of the study period, and thus their follow‐up was performed less than 6 months after the date of their operation. The remaining 248 (84.3%) mothers had their follow‐up performed 6 months after their procedure. During the follow‐up survey, 277 (94.2%) mothers reported no complaints. Among the 17 mothers who reported complaints, 11 (3.7%) described minor incisional pain and eight (2.7%) noted back pain (Table 4).

**TABLE 3:** Outcomes of mothers who were treated with the ESM‐Ketamine package and outcomes of newborns

Outcome (*n*, %)				
Label	Maternal outcome at discharge	Maternal outcome at 6 months	Newborn outcomes at discharge	Newborn outcome at 6 months follow up
Survival with no complaint	401 (100%)	277 (94.2%)	402 (92.4%)	300 (88.8%)
Death	0 (0%)	0 (0%)	33 (6.8%)	37 (10.9%)
Survival with complaint	0 (0%)	17 (5.7%)	0 (0%)	0 (0%)
Survival with disability	0 (0%)	0 (0%)	0 (0%)	1 (0.3%)
Follow‐up not possible	—	107	—	97

**TABLE 4:** Adverse events and complaints experienced by mothers who received the ESM‐Ketamine package

Adverse event during procedure	*n*, %
Hallucinations and/or agitation treated with diazepam	13 (3.2%)
Hypersalivation treated with atropine	63 (15.7%)
Peripartum hypertension treated with hydralazine	7 (1.7%)
Brief oxygen desaturations (SpO_2_ < 92% for <30 s)	33 (8.2%)

Serious adverse event during procedure	*n*, %
Respiratory arrest requiring cardiopulmonary resuscitation or bag‐valve mask ventilation	0 (0.0%)
Prolonged oxygen desaturations (SpO_2_ < 92% for >30 s)	0 (0.0%)

Complaint at 6‐month follow‐up	*n*, %
Minor incisional pain	11 (3.7%)
Constipation	1 (0.3%)
Dysmenorrhea	4 (1.4%)
Back pain	8 (2.7%)
Keloid	1 (0.3%)

A total of 402 (92.4%) of the 435 operative births who were delivered by ESM‐Ketamine supported emergency cesarean sections survived to discharge. Of the 33 births which did not survive to discharge, seven were macerated at birth, and three were missing death audits (making the cause of death unknown). The complications and conditions experienced by the remaining 23 are presented in Table 5, and detailed notes on each of these 23 cases are presented in Table S2. Among these 23, eight were in prolonged obstructed labor and were transferred from home or another facility that could not perform cesarean sections, eight had no fetal heart rate upon presentation for cesarean section, two had unstable fetal heart rates prior to cesarean section, two were actively hemorrhaging from abruption, four had severe intrauterine growth retardation, and one was septic. It should be noted that because the ESM‐Ketamine package was never the first‐choice approach, and was only employed when no anesthetist was available, the mothers had often been transferred from one, or to multiple facilities prior to receiving her emergency cesarean delivery. At 6‐month follow‐up, the status of 338 (77.7%) of the births could be confirmed, 300 (88.8%) of which were healthy with no complaints. Four newborns (1.2%) died of malaria or sepsis in the first 6 months after delivery, but no other deaths were reported during the follow‐up period. One newborn was diagnosed with partial blindness post‐discharge.

**TABLE 5:** Complications and conditions of the 23 non‐macerated births who did not survive to discharge

Complications and conditions	*n*, %
Prolonged obstructed labor	8 (34.7%)
No fetal heart rate (stillbirth)	8 (34.7%)
Severe intrauterine growth retardation	4 (17.4%)
Hemorrhage from placental abruption	2 (8.7%)
Unstable fetal heart rate at birth	2 (8.7%)
Birth asphyxia	6 (26.1%)
Sepsis	1 (4.3%)

There was no significant difference in newborn mortality at discharge and at 6‐month follow‐up (*P* = 0.821 and 0.834, respectively) between ESM‐Ketamine provider types (Table S3). All mothers survived to discharge and survived to 6‐month follow‐up, so there was also no difference in maternal mortality between ESM‐Ketamine provider types (Table S4).

## 4 DISCUSSION

In a cohort of 401 mothers, we found that the use of the ESM‐Ketamine package by trained non‐anesthetist providers working in rural hospitals in Kenya was both safe and effective in support of emergency cesarean operations. No serious maternal adverse events were reported, and 100% of mothers along with 92.4% of the births were healthy and without complaints at discharge. Moreover, at 6‐month follow‐up, the incidence of complications remained low, with 5.7% of mothers reporting minor complaints. These results suggest that non‐anesthetist mid‐level healthcare workers trained in the use of ketamine‐based anesthesia can significantly increase access to emergency cesarean delivery in resource‐poor settings when no anesthetist is available.

Access to cesarean section is a critical component of quality obstetric care, and immediate provision of this lifesaving procedure is vital to reducing maternal mortality and disability. However, shortages of trained anesthetists, a lack of essential equipment, and poor infrastructure make administration of modern anesthesia in low‐resource settings extremely challenging.^2^ Ketamine is a general anesthetic available worldwide and is a promising solution to enable cesarean delivery when no anesthetist is available.

Ketamine has been used as an anesthetic agent since the 1960s, and has numerous characteristics making it ideal for safe use in field settings.^12^ It is administered intravenously or intramuscularly, has minimal effects on central respiratory drive, produces bronchodilation, and unlike other anesthetics, does not depress the cardiovascular system.^9^ It is the anesthetic of choice for both the US and UK military when oxygen and monitoring supplies are limited.^9^ Furthermore, in the era of COVID‐19, the use of ketamine has further increased. It has become a preferred agent for deep procedural sedation to protect anesthetists and other providers from the risks associated with aerosolization from endotracheal intubation. The goal of the ESM‐Ketamine package was to create an effective, scalable intervention to support emergency cesarean delivery when no anesthetist is available.

To our knowledge, this study is the largest to characterize the use of ketamine for cesarean delivery. It includes 401 mothers, 435 newborns and stillbirths, nine healthcare facilities, and 54 mid‐level healthcare practitioners from various backgrounds. The findings of our analysis are promising and demonstrate that the ESM‐Ketamine package can be safely used by non‐anesthetist healthcare providers to facilitate cesarean delivery.

Although 401 consecutive emergency cesarean sections were safely supported by 54 trained non‐anesthetist providers in this case series, many of these providers supported a cesarean section so infrequently that it raises concerns about skill retention and quality of care if this were the only experience they are gaining. However, the ESM‐Ketamine providers over time expanded their use case to other emergency and urgent operative procedures when no anesthetist is available, such as in cases of open fractures, trauma, and laparotomies, among others.^10^ This expanded use enabled the ESM‐Ketamine providers to remain proficient in their technical skills over time. Moreover, because the ESM‐Ketamine package was used to support emergency general surgeries, orthopedic surgeries, and head/neck surgeries when no anesthetist was available, its impact extends far beyond its use in cesarean sections.

It has been suggested that ketamine may cause respiratory depression in newborns following cesarean delivery.^13^ Therefore it raises the question of whether in this cohort of 401 cesarean sections, ketamine contributed to the 33 births that did not survive to discharge. For this reason, each ketamine provider was trained in the Helping Babies Breathe protocol to immediately identify respiratory depression and provide bag‐valve mask ventilation when necessary. Furthermore, upon reviewing the 30 cases for which the causes of death were available, seven were macerated while the other 23 included home deliveries transferred multiple times while in obstructed labor, had severe intrauterine growth retardation, bradycardia, or no fetal heart rate at the time of cesarean section, among other complications (Table 5). These 23 would likely have benefitted from improved prenatal care and delivery at a facility capable of providing comprehensive emergency obstetrical care.

A recent analysis of over 50 000 in‐facility deliveries in Kenya and Uganda found that 89.8% of births survived to discharge.^14^ As these deliveries were facility‐based, we would expect a higher survival rate in this cohort than in the ESM‐Ketamine cohort, where mothers experienced obstructed deliveries and underwent late emergency cesarean sections. However, 92.4% of births in the ESM‐Ketamine cohort survived to discharge, supporting ketamine's strong safety profile in this population.

While ketamine is known to be a neuroprotective agent and has been demonstrated to maintain neonatal cerebral blood flow, few studies have extensively analyzed the impact of ketamine on neonatal development and physiology, and more research is required to fully characterize ketamine usage in neonates.^9^, ^15^ While WHO guidelines describe eclampsia, pre‐eclampsia, and hypertension as contraindications for ketamine use, ketamine administration in these groups has not been studied.^16^

This study did have a few limitations. It was conducted in one country (Kenya), and the majority of cases were treated at a single center (Sagam Sub‐County Hospital). However, the nine study sites do represent a broad range of contexts and included severely resource constrained facilities in settings of extreme poverty (refugee and nomadic), sub‐county community hospitals, and large county referral hospitals. These nine facilities are located in counties that have the highest burden of maternal mortality in Kenya.^17^ Furthermore, it should be noted that only 73.3% of mothers could be reached at 6‐month follow‐up. Many of the women belong to nomadic tribes or do not own phones, and as a result could not be contacted. Additionally, due to the prospective case series design of the study, comparisons between the efficacy of ketamine and standard general anesthesia could not be made.

Over the course of the past 7 years, the Kenyan Health system has been severely impacted by multiple prolonged healthcare worker strikes.^18^ These strikes resulted in significant reductions in hospital admissions and considerable health worker migration both within the country and out of the country. As a result, many trained ESM‐Ketamine providers were unable to utilize their skills, because the hospital they found themselves employed in could not support cesarean deliveries and other operative procedures.

In summary, this study provides evidence that use of the ESM‐Ketamine package by non‐anesthetist mid‐level providers in support of emergency cesarean sections may be safe and substantiates the immense promise of ketamine to increase access to lifesaving cesarean delivery in resource‐limited settings when no anesthetist is available. We acknowledge that anesthesia is best provided by specialty‐trained anesthetists who have access to necessary medicines, infrastructure support, and equipment, and the long‐term goal is to have skilled anesthetists available globally. However, this is not the current reality, and training many thousands of anesthetists will take decades while hundreds of thousands of mothers in resource‐poor settings suffer. The alternative to emergency cesarean surgery is often death and is particularly devastating as two lives are lost. It is immoral to deny mothers access to emergency anesthesia and life‐sustaining surgery simply because optimal anesthesia services are not available. Ketamine and other systems‐level innovations can play a major role in reducing maternal morbidity for underserved populations globally.

## CONFLICTS OF INTEREST

The authors have no conflicts of interest.

## AUTHOR CONTRIBUTIONS

Thomas Burke conceived of the ESM‐Ketamine package and this study, wrote, and edited the manuscript and assumes overall responsibility of the study. Sreekar Mantena extracted the data, led data analysis, and took lead in writing the manuscript. Kennedy Opondo led data collection and extraction and provided manuscript edits. Solomon Orero and Khama Rogo have been important senior advisors throughout the research efforts, and both reviewed the manuscript and provided edits. All authors approved the final manuscript draft.

## Supporting information

Table S1‐S4
