# Supplementary material for: A ketamine package for use in emergency cesarean delivery when no anesthetist is available: An analysis of 401 consecutive operations
Source: Int J Gynaecol Obstet. 2021 Oct 28;158(2):377–84. doi: 10.1002/ijgo.13965 (PMC9545139; doi:10.1002/ijgo.13965)
Supplement: Supplementary file 1 — Table S1‐S4 [file IJGO-158-377-s001.docx]

**SUPPLEMENTARY TABLES**

Table S1: Characteristics of participating facilities.

| **Hospital Name** | **Type of Facility** | **County** | **County poverty level** | | | | | **Number of hospital beds** | | **Number of ESM-Ketamine trained providers** | **Number of anesthetists** | **Number of operating theatres** | **Number of Annual Deliveries** | | **Estimated population of hospital's catchment area** |
| --- | --- | --- | --- | --- | --- | --- | --- | --- | --- | --- | --- | --- | --- | --- | --- |
|  |  |  | Children <18 yrs | Youths (18-34 yrs) | Adults (35-59 yrs) | Elderly (60+ yrs) | Total Population | General | Maternity |  |  |  | Normal Delivery | Cesarean Delivery |  |
| Sagam Community Hospital | Private | Siaya | 65.20% | 70.10% | 82.90% | 62.70% | 68.80% | 100 | 8 | 5 | 2 | 1 | 329 | 140 | 5021 |
| Maseno Mission Hospital | Private | Kisumu | 27.60% | 39.40% | 52.90% | 35.10% | 35.50% | 36 | 10 | 3 | 2 | 1 | 537 | 78 | 10502 |
| Muhoroni Sub-county Hospital | Public | Kisumu | 27.60% | 39.40% | 52.90% | 35.10% | 35.50% | 44 | 20 | 3 | 2 | 1 | 603 | 72 | 15829 |
| Kendu Sub-County Hospital | Public | Homa Bay | 76.10% | 80.10% | 83.00% | 84.40% | 78.40% | 30 | 12 | 2 | 1 | 1 | 1285 | 92 | 19779 |
| Mbita Sub-County Hospital | Public | Homa Bay | 76.10% | 80.10% | 83.00% | 84.40% | 78.40% | 86 | 26 | 2 | 2 | 1 | 960 | 60 | 7374 |
| Dadaab Sub-County Hospital | Public | Garissa | 66.20% | 71.00% | 76.50% | 72.80% | 69.00% | 100 | 30 | 2 | 3 | 1 | 287 | 118 | 12000 |
| Modogashe Sub-County Hospital | Public | Garissa | 66.20% | 71% | 76.50% | 72.80% | 69% | 25 | 5 | 1 | 1 | 1 | 217 | 3 | 17515 |
| Ijara Sub-County Hospital | Public | Garissa | 66.20% | 71% | 76.50% | 72.80% | 69% | 24 | 6 | 1 | 1 | 1 | 517 | 39 | 9743 |
| Takaba Sub-County Referral Hospital | Public | Mandera | 90.20% | 93.40% | 94.10% | 92.60% | 91.50% | 50 | 50 | 4 | 4 | 1 | 650 | 72 | 20125 |

Table S2: Characteristics of the 23 non-macerated births who did not survive to discharge

| **Mother #** | **Mother Age (yrs)** | **Dose of Ketamine (mg/kg)** | **Complications Experienced During Pregnancy or Delivery** |
| --- | --- | --- | --- |
| 1 | 24 | 15.0 | Placental abruption while laboring at home |
| 2 | 21 | 18.0 | Obstructed labor at home in premature fetus. Fetal heart rate undetectable |
| 3 | 17 | 5.0 | Obstructed labor in premature fetus. Referred from facility unable to perform cesarean section |
| 4 | 22 | 5.2 | Pre-eclampsia and severe malaria with premature fetus |
| 5 | 26 | 6.0 | Severe intrauterine growth retardation with obstructed labor from brow presentation |
| 6 | 24 | 6.0 | Intrauterine growth retardation and umbilical cord prolapse |
| 7 | 22 | 6.0 | Obstructed twin birth referred from facility unable to perform cesarean section. Irregular fetal heart rate |
| 8 | 25 | 5.8 | Obstructed labor and brow presentation referred from facility unable to perform cesarean section |
| 9 | 20 | 6.0 | Intrauterine growth retardation and birth asphyxia |
| 10 | 26 | 3.5 | Birth asphyxia |
| 11 | 24 | 4.0 | Pre-eclampsia and obstructed labor at home. No fetal heart tones obtainable |
| 12 | 27 | 8.0 | Obstructed labor and premature birth referred from facility unable to perform cesarean section. Irregular fetal heart rate |
| 13 | 28 | 8.0 | Obstructed labor with intrauterine growth retardation of premature fetus with no fetal heart rate obtainable |
| 14 | 15 | 6.0 | Obstructed labor with severe hemorrhage |
| 15 | 30 | 2.0 | Obstructed labor referred from facility unable to perform cesarean section with no fetal heart rate obtainable |
| 16 | 23 | 2.7 | Reduced fetal activity and no obtainable fetal heart rate |
| 17 | 17 | 3.0 | Fresh still birth, no obtainable fetal heart rate |
| 18 | 24 | 3.7 | Fresh still birth, no obtainable fetal heart rate |
| 19 | 33 | 5.0 | Birth asphyxia |
| 20 | 27 | 4.0 | Birth asphyxia |
| 21 | 20 | 3.0 | Birth asphyxia |
| 22 | 26 | 6.0 | Birth asphyxia |
| 23 | 22 | 3.9 | Fresh still birth, no obtainable fetal heart rate |

Table S3: Newborn outcomes stratified by ESM-Ketamine provider type

| **Newborn Outcomes at Discharge (n, %)** | | | |
| --- | --- | --- | --- |
|  | Nurse-Midwife | Medical Officer | Clinical Officer |
| Survival | 169 (91.8%) | 18 (90.0%) | 215 (93.1%) |
| Survival with disability | 0 | 0 | 0 |
| Death | 15 (8.2%) | 2 (10.0%) | 16 (6.9%) |
| **Newborn Outcomes at 6 Month Follow-Up (n, %)** | | | |
|  | Nurse-Midwife | Medical Officer | Clinical Officer |
| Survival | 136 (73.0%) | 17 (75.0%) | 147 (62.4%) |
| Survival with disability | 0 (0%) | 0 (0%) | 1 (0.5%) |
| Death | 16 (7.9%) | 3 (15.0%) | 18 (7.1%) |
| Follow-up not possible | 32 (19.1%) | 0 (0%) | 65 (30.0%) |

Table S4: Maternal outcomes stratified by ESM-Ketamine provider type

| **Maternal Outcomes at Discharge (n, %)** | | | |
| --- | --- | --- | --- |
|  | Nurse-Midwife | Medical Officer | Clinical Officer |
| Survival | 168 (100%) | 19 (100%) | 214 (100%) |
| Survival with complaint | 0 (0%) | 0 (0%) | 0 (0%) |
| Death | 0 (0%) | 0 (0%) | 0 (0%) |
| **Maternal Outcomes at 6 Month Follow-Up (n, %)** | | | |
|  | Nurse-Midwife | Medical Officer | Clinical Officer |
| Survival | 128 (75.7%) | 18 (94.7%) | 131 (61.5%) |
| Survival with complaint | 7(4.1%) | 1 (5.3%) | 9 (4.2%) |
| Death | 0 (0%) | 0 (0%) | 0 (0%) |
| Follow-up not possible | 34 (20.1%) | 0 (0%) | 73 (34.3%) |
